# The relationship between fear of cancer recurrence and death anxiety among Chinese cancer patients: the serial mediation model

**DOI:** 10.1186/s12888-024-05819-8

**Published:** 2024-06-04

**Authors:** Furong Chen, Meijun Ou, Zhirui Xiao, Xianghua Xu

**Affiliations:** 1 The Affiliated Cancer Hospital of Xiangya School of Medicine, Central South University/Hunan Cancer Hospital, Changsha, Hunan 410013 China; 2 School of Nursing, University of South China, Hengyang, Hunan 421001 China

**Keywords:** Fear of cancer recurrence, Meaning in life, Experiential avoidance, Death anxiety, Serial mediation model

## Abstract

**Aims:**

This study aims to investigate the association between fear of cancer recurrence (FCR) and death anxiety (DA) among Chinese cancer patients, while considering the mediating effects of experiential avoidance (EA) and meaning in life (MIL).

**Methods:**

From February to June 2023, convenience sampling was used to select newly diagnosed cancer patients in a tertiary Cancer Hospital in Chinese Hunan Province as the survey objects. A total of 436 cancer patients completed the Fear of Cancer Recurrence Inventory, the Meaning in Life Questionnaire, the Acceptance and Action Questionnaire-II, and the Templer’s death anxiety scale. Descriptive analysis and Pearson correlation analysis were conducted using SPSS 28.0 software. Serial mediation analysis was performed by Hayes’ PROCESS macro.

**Results:**

Gender, age, educational level, marital status, residence, occupation, per capita monthly household income, tumor type, and cancer stage were controlled in the model. The results revealed that fear of cancer recurrence had a significant direct effect on death anxiety (Effect = 0.075, 95% CI: 0.064 to 0.087). Additionally, three indirect pathways were identified: (1) through experiential avoidance (Effect = 0.037, 95% CI: 0.026 to 0.049), (2) through meaning in life (Effect = 0.022, 95% CI: 0.014 to 0.031), and (3) through the serial mediators involving meaning in life and experiential avoidance (Effect = 0.016, 95% CI: 0.010 to 0.023). The total indirect effect of the three mediation paths was 63.56%.

**Conclusion:**

Fear of cancer recurrence is a significant psychological distress experienced by cancer patients, which not only directly contributes to death anxiety but also may triggers changes, such as experiential avoidance and meaning in life. Ultimately, this comprehensive psychological distress leads to death anxiety.

## Introduction

Over the past decade, cancer incidence and mortality rates among the Chinese have increased at an annual rate of approximately 3.9% and 2.5% [1]. By the time most cancer patients were newly diagnosed, they were already in the middle or late stage, losing the best chance of treatment [2], becoming the group closest to death. When cancer patients bear the burden of the disease, they are always worried about the progression and recurrence of the disease and face the threat of death, which generates a series of negative emotions, including fear of cancer recurrence (FCR) and death anxiety (DA) [3, 4].

Fear of cancer recurrence is defined as “Fear, worry or concern relating to the possibility that cancer will come back or progress” [5], which is one of the most commonly recognized unmet psychological needs of cancer patients [6]. Patients with high FCR were more likely to have difficulty controlling frequent distressing thoughts, to believe more strongly that cancer would return and be able to describe more detailed thoughts related to death, and to experience anxiety and fear in the process [7, 8]. DA is defined as “emotional reaction provoked by anticipation of death generated by perceptions of areal or imaginary threat to the existence of one’s own or people close to them”. This diagnosis was incorporated into Domain 9—Stress Coping/tolerance by the North American Nursing Diagnosis Association (NANDA) to guide the clinical judgment for nurses [9]. Some scholars have speculated that is possible that DA is another manifestation of FCR [10]. We further need studies to investigate the role of FCR in DA, by firstly confirming the proposed relationship between the two constructs.

In explaining individual responses to cancer, Curran’s cancer-related anxiety model [11] proposes that preexisting vulnerability factors, contextual factors, cognitive content, and coping responses affect FCR. The model places greater emphasis on existential factors such as disruption of core beliefs, perception of the meaning in life, and fear related to death. In particular, the model emphasizes that preexisting schema about the self, relationships, or the world may be questioned following a cancer diagnosis, creating feelings of instability and vulnerability such as death anxiety. Conversely, psychological distress could be reduced when a person has meaning and purpose despite having cancer [12]. The model also suggests that DA is more likely to stem from a sense of destruction in the worldview and a lack of meaning and purpose in life, which is likely fueled by fear of cancer recurrence.

From the core of this theory, it is apparent that the content of death-related anxiety, such as concerns about the manner of one’s death, the impact of one’s death on others, or what will happen after death, is expected to overlap with concerns about disease recurrence or progression. FCR is a fear response to cancer recurrence caused by specific things and events related to cancer, which has specificity and individuality. DA is related to human core values and individual beliefs, which is more abstract and universal. Both FCR and DA are unresolved psychological problems in cancer patients, and the relationship between them may be normalized from specificity to universality which needs further exploration. In particular, whether the FCR will transform into DA, and how to implement the corresponding mental nursing measures.

The response of cancer patients to the diagnosis, treatment, and recurrence of cancer is usually experiential avoidance (EA) [13], which is manifested as trying to avoid or ignore unpleasant thoughts, feelings, and painful memories, which violates personal values and causes long-term damage [14], and often affects their quality of life, especially results in the negative emotions such as anxiety and depression [15]. The equivalent of that, EA has been highlighted as a risk factor for psychopathology and mental health deterioration. For example, among various psychological disorders, individuals with anxiety and major depression have higher levels of EA [16, 17]. In addition, it has also been shown that EA can affect DA levels in Chinese cancer patients [18]. Although negative coping strategies such as avoidance can alleviate DA in the short term, may be detrimental to general health and quality of life in the long run [19]. Therefore, patients with fear of cancer recurrence may experience death anxiety after adopting experiential avoidance strategies.

Meaning in life (MIL) is one of the psychological domains most profoundly impacted in terminally ill patients [20]. The presence of MIL can enhance patients’ sense of happiness, promote coping mechanisms, and improve their ability to tolerate physical symptoms [21]. Furthermore, MIL can potentially serve as a protective buffer for cancer and terminal patients, helping to prevent the development of depression, despair, and the generation of death wishes [22, 23]. When individuals undergo the process of cancer diagnosis, treatment, and recovery, they often experience deep worry and fear of cancer recurrence, which may prompt them to reevaluate their lives, values, and the overall meaning of their existence. These symbolic concerns may be connected to more intense FCR. FCR may lead to an increase of death anxiety by reducing the sense of meaning in life. The most influential Terror Management Theory (TMT) [24] of death anxiety holds that when individuals perceive the threat of death, they employ a variety of defense mechanisms - such as reaffirming their worldview, seeking meaning in life, bolstering self-esteem, and strengthening close relationships - to alleviate the resulting death anxiety. In the empirical study, Neel et al. [25] found a negative correlation between MIL and death anxiety among patients with advanced cancer.

A negative relationship between experiential avoidance and meaning in life has been reported [13]. As suggested by Hayes et al. [26], experiential avoidance encompasses coping behaviors that involve avoiding distressing thoughts, emotions, and feelings, thereby diminishing the patient’s perception of life. This may weaken the creation and experience of patients’ sense of meaning in life. Moroz and Dunkley [27] found that experiential avoidance may lead to a lack of meaning in life creation in patients while predicting an increase in depressive and anxiety symptoms [17]. Additionally, individuals who possess significant life goals are less inclined to engage in avoidance behaviors compared to those without such goals [28]. The aforementioned research and summary highlight that when cancer patients experience fear of cancer recurrence, they may often adopt the coping strategy of experiential avoidance, which weakens their sense of meaning in life and leads to an increase in death anxiety. It is possible, therefore, that experiential avoidance and meaning in life serve as mediators in the relationship between fear of cancer recurrence and death anxiety.

## Current study

**Fig. 1:**
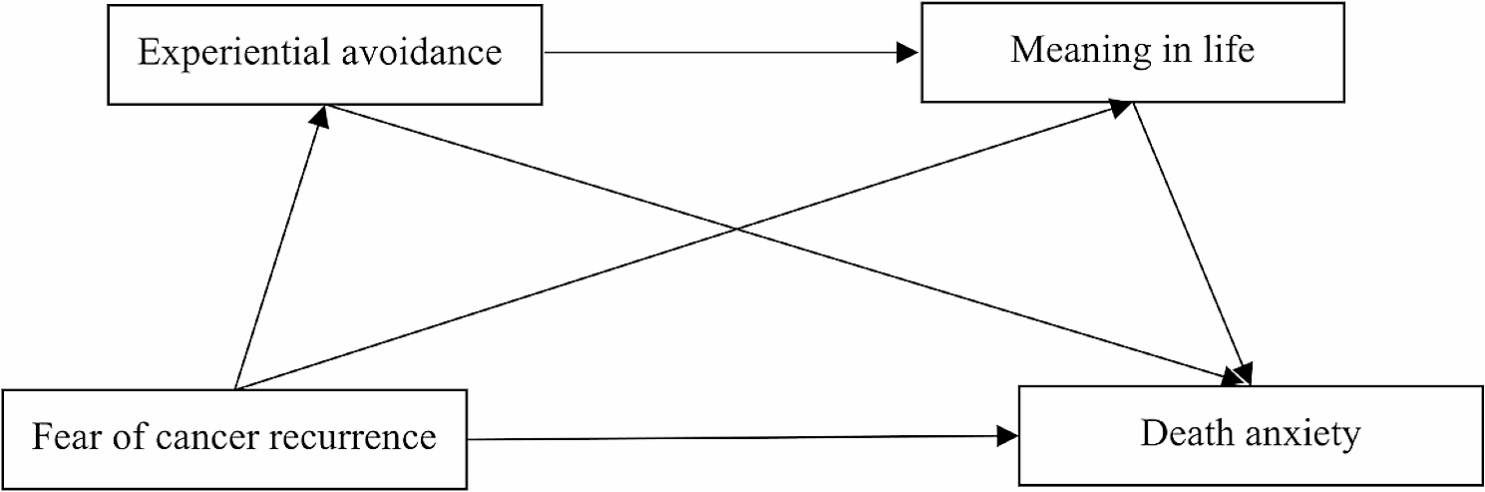
The proposed serial mediation model

By delving into the underlying psychological mechanisms of the relationship between fear of cancer recurrence and death anxiety, we can potentially minimize the adverse effects of these events on the psychological status of cancer patients. This exploration has important theoretical and practical implications. However, no studies have focused on the possible coexistence of experiential avoidance and meaning in life, how they interact with each other and impact on death anxiety in Chinese samples of newly diagnosed cancer patients. This study believes that the relationship between fear of cancer recurrence and death anxiety may be serially mediated by the two mediators of experiential avoidance and meaning in life. In summary, the current study aims to test a serial mediation model linking FCR to death anxiety among Chinese newly diagnosed cancer patients through experiential avoidance and meaning in life. The current study offers the following four hypotheses (Fig. 1):

### H_1_

Fear of cancer recurrence is positively correlated with death anxiety.

### H_2_

Experiential avoidance mediates the link between fear of cancer recurrence and death anxiety.

### H_3_

Meaning in life mediates the link between fear of cancer recurrence and death anxiety.

### H_4_

Experiential avoidance and Meaning in lifesequentially mediate the link between fear of cancer recurrence and death anxiety.

## Methods

### Participants

This study was a cross-sectional survey. From February to June 2023, convenience sampling was used to select cancer patients in a tertiary Cancer Hospital in Chinese Hunan Province as the survey objects. Candidates eligible to participate in this study were cancer patients with newly diagnosed malignant tumors confirmed by pathological results within 7 days, aged 18 years or older, whose cognitive function was normal, and knew their diagnosis, who also volunteered to participate in this study. Patients with existing or previous mental diseases, psychological disorders, communication or reading comprehension problems, or inability to cooperate due to severe illness, and cancer patients who experience the presence of major diseases other than cancer were not allowed to participate in this study.

The G*Power package program was used to calculate the sample size [29]. We chose the Correlation: Point biserial model in the t-test for analysis and used the two-tailed test with the Effect size set to 0.2, significant level (α) err prob set to 0.01. Taking the statistical power (1-β err prob) = 0.95. The final sample size was calculated as 431 cases.

The researchers screened patients from outpatients and inpatients who met the inclusion criteria, and the final included patients signed an informed consent form after agreeing to participate in the survey. Under the premise of not disturbing the patients’ treatment or rest, the researcher used paper questionnaires to conduct one-on-one surveys for the patients. Upon completion of the questionnaire, the researchers meticulously examined for any missing information. In cases where queries arose, the researchers promptly sought clarification from the respondents. All questionnaires were distributed and collected on-site. A total of 465 questionnaires were distributed, yielding 436 valid responses, resulting in an impressive recovery rate of 92.04%. Among them, 16 questionnaires were interrupted, and 13 were invalid (all scales were repeatedly checked).

### Measures

#### Basic characteristics

The basic characteristics of the participants including gender, age, educational level, marital status, residence, occupation, per capita monthly household income, tumor type, and cancer stage, were collected.

#### The fear of cancer recurrence inventory

The fear of cancer recurrence inventory (FCRI) was developed by Savard et al. [30] and used to assess fear of cancer recurrence symptoms. FCRI assessed 42 items in 7 dimensions, including trigger factors (8 items), severity (9 items), psychological distress (4 items), dysfunction (6 items), coping strategies (9 items), insight (3 items), and seeking comfort (3 items). Likert 5 score was adopted, and item 13 was the inverted score. The higher the score, the higher the FCR. The total Cronbach’s α was 0.96, and the retest reliability was 0.88 [31]. The Chinese version of FCRI [32] was used in this study and its Cronbach’s *α* was 0.83.

#### The Templer’s death anxiety scale

This scale was developed by Templer et al [33]. and is the most commonly used scale to evaluate the degree of death anxiety in cancer patients. There were 15 items in total, including 4 dimensions of cognition, emotion, time awareness, stress and pain. The higher the total score, the more serious the degree of death anxiety. The total score ≥ 7 indicates the existence of death anxiety and the Cronbach α coefficient of the scale was 0.867, and the test-retest reliability was 0.831 [34]. The Chinese version of this scale [35] was used and the Cronbach’s *α* in this study was 0.88.

#### The second edition of the acceptance and action questionnaire-II

The second edition of the Acceptance and Action Questionnaire-II(AAQ-II) was compiled by Bond [36]. It was a commonly used tool for evaluating EA [14], with 7 items, ranging from “never” to “always”, and the total score of the 7 items was added up. The total Cronbach’s α coefficient of the scale was 0.88 [14]. The Chinese version of AAQ-II [37] was used in the current study and Cronbach’s *α* was 0.86.

#### The meaning in life questionnaire

The scale was compiled by Steger [38] in 2006, including two dimensions: the sense of meaning of life (5 items) and the search for the sense of meaning of life (4 items). Each item was assigned a score of 1 to 7 from “strongly disagree” to “strongly agree” on a scale of 10 to 70, with a higher score indicating a stronger sense of meaning in life. The Cronbach’s α coefficient was 0.71 [38]. The Chinese version of this scale [39] was used in this study and the Cronbach’s α was 0.85.

### Statistical analysis

We used the Statistical Package for the Social Sciences (SPSS) 26.0 software and PROCESS 4.1 for data analysis. Firstly, the variance inflation factor (VIF) was used to assess multi-collinearity, and the measurement data were described by means and standard deviations or frequency and percentages. Second, Pearson correlation analysis was conducted to examine the relationships among four variables: fear of cancer recurrence, experiential avoidance, meaning in life, and death anxiety. Subsequently, we examined the chain mediating role of experiential avoidance and meaning in life between fear of cancer recurrence and death anxiety in Chinese cancer patients using the PROCESS Model 6 by Andrew F. Hayes [40]. In addition, to evaluate the impact of fear of cancer recurrence on death anxiety, we calculated 95% confidence intervals through bias-corrected percentile bootstrapping using a sample size of 5000 [41]. Gender, age, educational level, marital status, residence, occupation, per capita monthly household income, tumor type, and cancer stage were controlled in the model. Statistical significance was determined by a two-tailed p-value below 0.05.

## Results

### Testing for multi-collinearity

Collinearity diagnostics showed the variance inflation factor for fear of cancer recurrence, experiential avoidance, meaning in life was 2.132, 3.135, 2.763, respectively, which was below the threshold of 5 [42]. Thus, the multi-collinearity might not affect our estimates.

### The demographic characteristics of the participants

The 436 cancer patients investigated in this study were 19 to 80 (7.27 ± 11.35) years old, and the remaining general data were shown in Table 1.

**Table 1:** Demographic characteristics of the participants (*N* = 436)

Variables	*N*	%
Gender		
Male	221	50.69
Female	215	49.31
Age (years)		
≥ 18	182	41.74
≥ 45	212	48.62
≥ 60	42	9.64
Educational level		
Primary school or below	109	25.00
Middle school	239	54.82
College/bachelor’s degree	71	16.28
Master’s degree or above	17	3.90
Marital status		
Married	376	86.24
Unmarried	33	7.57
Divorced/widowed	27	6.19
Residence		
Urban	215	49.31
Rural	221	50.69
Occupation		
Public official	108	24.77
Worker	94	21.56
Farmer	131	30.05
Self-employed	47	10.78
Unemployed	36	8.26
Retired	20	4.58
Per capita monthly household income(RMB)		
< 3000	114	26.15
≥ 3000	149	34.17
≥ 5000	120	27.52
≥ 10,000	53	12.16
Tumor type		
Head and neck cancer	75	17.20
Breast cancer	56	12.84
Pulmonary tumors	72	16.51
Digestive system neoplasms	83	19.04
Urinary system neoplasms	86	19.72
Bone and soft tissues malignant tumors	64	14.69
Cancer stage		
I	75	17.20
II	148	33.94
III	126	28.89
IV	87	19.97

### Pearson’s correlation analysis

The results of the mean, standard deviation, and correlation coefficient of the variables in this study were shown in Table 2. The results of correlation analysis showed that fear of cancer recurrence was significantly positively correlated with death anxiety (*r* = 0.820, *p* < 0.001) and, experiential avoidance (*r* = 0.788, *p* < 0.001) but negatively correlated with meaning in life (*r*=-0.755, *P* < 0.001). While experiential avoidance was significantly negatively correlated with meaning in life (*r*=-0.785, *p* < 0.001). Moreover, we also found death anxiety was significantly positively correlated with experiential avoidance (*r* = 0.829, *p* < 0.001), and negatively correlated with meaning in life (*r*=-0.832, *p* < 0.001).

**Table 2:** Descriptive statistics and correlations of the study variables (*N* = 436)

Variables	M	SD	FCR	EA	MIL	DA
FCR	89.134	30.442	1^***^			
EA	26.758	11.754	0.788^***^	1^***^		
MIL	33.275	11.914	-0.75^***^	-0.785^***^	1^***^	
DA	8.148	4.646	0.820^***^	0.829^***^	-0.832^***^	1^***^

*Note* M = mean; SD = standard deviation, FCR: Fear of cancer recurrence, EA: Experiential avoidance, MIL: Meaning in life, DA: Death anxiety; ^***^P < 0.001

### Testing serial mediation model

**Fig. 2:**
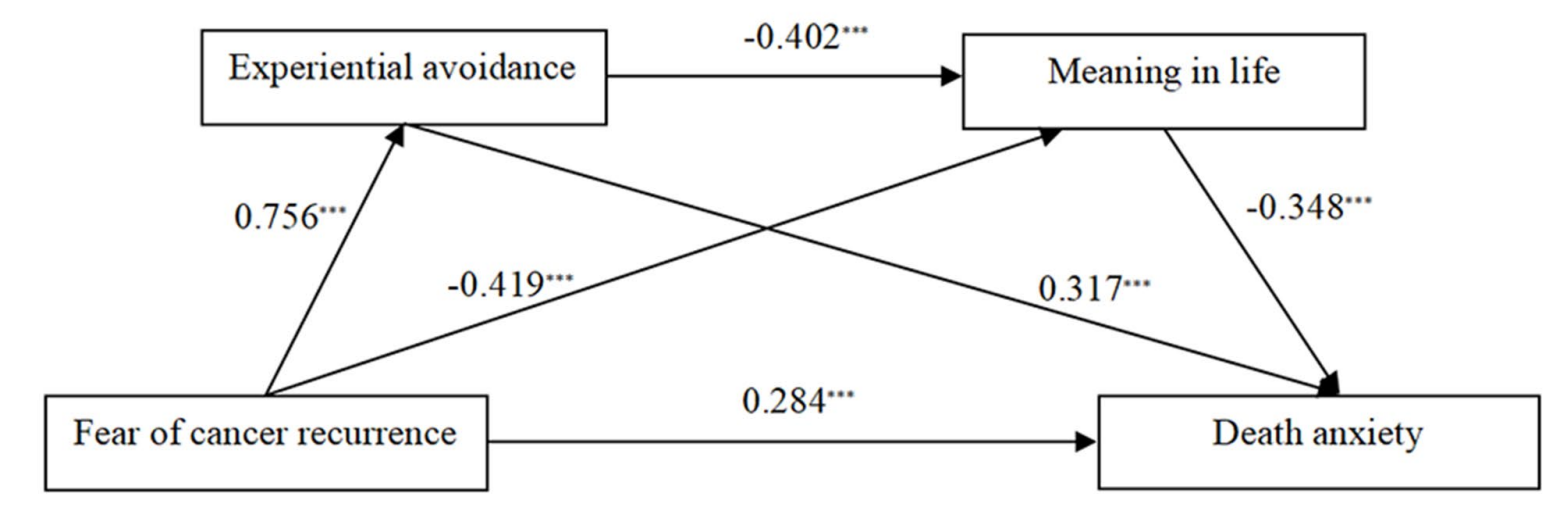
The serial mediation model of experiential avoidance and meaning in life in the relationship between fear of cancer recurrence and death anxiety. Note^***^P < 0.001

Model 6 in SPSS PROCESS macro was used to test the serial mediation effect. The regression-based analysis results were shown in Table 3. As shown, fear of cancer recurrence was positively associated with experiential avoidance(β = 0.756, *p* < 0.001), which, as well with death anxiety(β = 0.317, *p* < 0.001); Meanwhile, the direct effect of fear of cancer recurrence on death anxiety was significant (β = 0.284, *p* < 0.001). Thus, Hypothesis 1 and 2 were confirmed. Moreover, consistently with Hypothesis 3, fear of cancer recurrence was negatively associated with meaning in life(β = -0.419, *p* < 0.001), which, in turn, meaning in life was negatively associated with death anxiety(β =−0.348, *p* < 0.001). Additionally, experiential avoidance was negatively associated with meaning in life(β = -0.402, *p* < 0.001), forming a serial mediation effect, which supported Hypothesis 4. The serial mediation model was presented in Fig. 2.

**Table 3:** Regression-based results in the serial mediation analysis (*N* = 436)

Criterion	Predictors	*R*	*R* ^2^	F	coefficients	β	t	95% CI
EA	FCR	0.804	0.646	73.765	0.292^***^	0.756	23.678	0.267 ~ 0.316
MIL	FCR	0.810	0.656	70.047	-0.164^***^	-0.419	-8.639	-0.202~-0.127
	EA				-0.408^***^	-0.402	-8.207	-0.505~-0.310
DA	FCR	0.906	0.821	153.913	0.043^***^	0.284	7.416	0.032 ~ 0.055
	EA				0.125^***^	0.317	8.278	0.095 ~ 0.155
	MIL				-0.136^***^	-0.348	-9.681	-0.163~-0.108

### Bootstrap test of mediating effect

Bootstrap test with 5000 bias-corrected bootstrapping samples was used to examine the statistical significance of indirect effects. Table 4 showed that experiential avoidance and meaning in life partially mediated the relationship between fear of cancer recurrence and death anxiety, with a total indirect effect of 0.075, accounting for 63.56% of the total effect. Specifically, fear of cancer recurrence indirectly affected death anxiety through three significant mediating pathways: (1) experiential avoidance (*effect* = 0.037, 95*% CI*: 0.026 to 0.049), accounting for 31.36% of the total effect; (2) meaning in life (*effect* = 0.022, 95% *CI*: 0.014 to 0.031), accounting for 18.64% of the total effect; and (3) the serial mediators involving in experiential avoidance and meaning in life(*effect* = 0.016, 95% *CI*: 0.010 to 0.023), which accounted for 13.56% of the total effect.

**Table 4:** Fear of cancer recurrence and death anxiety in the mediation effect analysis (*N* = 436)

Effect	Effect size	Boot SE	Boot LLCI	Boot ULCI
Total effects	0.118	0.004	0.109	0.127
Direct effect	0.043	0.006	0.032	0.055
Total indirect effect	0.075	0.006	0.064	0.087
Indirect effect (X → M1 → Y)	0.037	0.006	0.026	0.049
Indirect effect (X → M2 → Y)	0.022	0.004	0.014	0.031
Indirect effect (X → M1 → M2 → Y)	0.016	0.003	0.010	0.023

*Note* Based on 5000 bootstrap samples; Total, direct, and indirect effects of fear of cancer recurrence (X) on death anxiety (Y) through experiential avoidance (M1) and meaning in life (M2); SE = standard error; CI = confidence interval

Moreover, the direct effect of fear of cancer recurrence on death anxiety remained significant. This serial mediation model accounted for 82.1% of the variance in death anxiety among cancer patients (*F* = 153.913, *p* < 0.001). Thus, experiential avoidance and meaning in life played partially mediating roles in the relationship between fear of cancer recurrence and death anxiety.

## Discussion

Despite mounting evidence suggesting that the fear of cancer recurrence can significantly impact death anxiety [10, 43], hardly any studies have investigated the role of experiential avoidance and meaning in life in this relationship using a process-oriented approach. Building upon previous research and theoretical support, we constructed a serial mediation model. We found that fear of cancer recurrence significantly affected death anxiety, and the relationship between them was mediated in part by experiential avoidance and meaning in life. These findings hold promise for the development of targeted psychological interventions aimed at mitigating the risk of death anxiety in cancer patients who exhibit heightened levels of experiential avoidance and diminished sense of meaning in life.

We discovered that experiential avoidance serves as a mediator in the relationship between fear of cancer recurrence and death anxiety. Specifically, heightened levels of fear of cancer recurrence were found to be associated with increased experiential avoidance, which, in turn, correlated with elevated levels of death anxiety. According to TMT [44], awareness of death or the perception of its proximity can elicit unbearable anxiety due to humanity’s innate survival instinct. Therefore, when faced with the possibility of cancer recurrence, individuals often resort to proximal defense mechanisms, such as avoidance [45, 46], in an attempt to alleviate death anxiety [47]. This is an understandable response that is often effective in reducing anxiety in the short term. But in the long run, avoidance would increase psychological distress of them, trigger deeper death-related thinking, and feel anxious about death-related results. Cancer patients should strive to cultivate an accepting mindset to mitigate the impact of fear of recurrence on death anxiety. On the other hand, experiential avoidance accounted for 31.36% of the total effect, which was obviously beyond the total effect occupied by meaning in life. This may be due to the fact that when patients are faced with the newly diagnosed of cancer, the first measure they often take is to deny it, and after subsequent treatment, they may tend to find meaning in life, which is also in line with the core concept of TMT. In conclusion, experiential avoidance triggered by fear of cancer recurrence emerges as a significant contributing factor in the development of death anxiety.

In our study, we observed a negative correlation between meaning in life and fear of cancer recurrence, marking the first finding in the psychological realm concerning cancer patients. Additionally, we found a negative association between meaning in life and death anxiety, which aligns with previous research findings [25, 48]. These results provide support for our hypothesis that meaning in life serves as a mediator in the relationship between fear of cancer recurrence and death anxiety. Liu et al. [48] demonstrated that cancer patients with a diminished sense of meaning in life were more susceptible to developing death anxiety. Specifically, fear of cancer recurrence emerges as a significant risk factor for death anxiety among cancer patients who have a low sense of meaning in life. Meaning in life establishes an adaptive defense mechanism against death anxiety by fostering a positive value orientation rooted in individuals’ acceptance of mortality and their commitment to expanding and enriching their lives through self-transcendence [49]. This implies that meaning in life, as a protective factor for mental well-being, empowers cancer patients to maintain resilience in the face of potential cancer recurrence. Cancer patients who possess a high level of meaning in life can mobilize a positive mindset when confronting cancer treatments and follow-up examinations. They can alleviate death anxiety resulting from fear of cancer recurrence through their optimistic beliefs in anti-cancer treatments and a strong sense of self-worth. Such individuals are capable of seeking personal value and finding solace amidst the challenges posed by cancer, thereby enhancing their adaptability and overall mental well-being.

As hypothesized, experiential avoidance and meaning in life sequentially mediate the link between fear of cancer recurrence and death anxiety. This study aligns with previous research, which has also documented a negative correlation between experiential avoidance and meaning in life [17]. It is also worth mentioning that the serial mediators effect (13.56%) was smaller than the total mediating effect (63.56%) in this study, which may be because the death anxiety of cancer patients is more likely to be affected by empirical avoidance or meaning in life alone. Drawing upon the avoidance model of worry [50], it is evident that cancer patients who experience concerns about recurrence and symptoms suggestive of recurrence tend to engage in mental imagery avoidance, deliberately evading thoughts and anxieties related to the potential progression of the disease leading to death. This persistent avoidance of distressing thoughts among cancer patients may create a discordance with their aspirations and values [51], ultimately culminating in a diminished sense of meaning in life. These factors collectively contribute to heightened levels of anxiety surrounding death. Consequently, cancer patients who lack a profound sense of meaning face greater challenges in comprehending the purpose of life, interpreting life’s essence through the lens of their values, clarifying self-directed goals, and objectively confronting mortality.

### Limitations and future directions

Our study provides novel evidence of a serial mediating effect of experiential avoidance and meaning in life in the relationship between fear of cancer recurrence and death anxiety. Nonetheless, it is important to acknowledge several limitations inherent in our study. Firstly, due to its cross-sectional design, establishing causal relationships among these variables is challenging. Therefore, future research should prioritize large-scale, multi-center prospective studies to validate and expand upon our findings. Secondly, it is crucial to exercise caution when generalizing the results of this study, as the questionnaire survey was exclusively conducted at a Chinese single tertiary Cancer Hospital in Hunan Province. Thus, the cultural and contextual factors specific to this setting may influence the generalizability of our findings. Thirdly, while we took measures to mitigate common method bias, as evidenced by the analysis, it is essential to acknowledge the potential presence of subjective bias and social desirability effects inherent in self-rated questionnaires. Lastly, our study did not account for psychiatric comorbidities that may impact fear of cancer recurrence and death anxiety, such as depression and loneliness among cancer patients. Future research endeavors should consider incorporating these comorbidities into the model to enhance its comprehensiveness and effectiveness.

### Practical implications

Despite these limitations, our study holds important practical implications. The common use of experiential avoidance strategies and the low sense of meaning in life among cancer patients may contribute to heightened levels of death anxiety in individuals with fear of cancer recurrence. Consequently, it is imperative to consider targeted interventions that address both experiential avoidance and the cultivation of meaning in life as means to alleviate psychological distress in this population. To the best of our knowledge, acceptance and commitment therapy (ACT) may be an effective psychological intervention for reducing fear of recurrence and death anxiety in cancer patients. ACT focuses on diminishing experiential avoidance while encouraging patients to clarify their purpose and meaning in life, ultimately inspiring them to commit to practical actions aligned with their self-worth [52]. Therefore, we suggest that nursing professionals develop and implement prevention and intervention programs that target experiential avoidance and meaning in life, such as ACT, to promote and sustain the psychological well-being of cancer patients. By addressing these crucial aspects, nursing providers can enhance the overall quality of care and support provided to individuals navigating the challenges of cancer.

## Conclusion

In conclusion, this study provides significant empirical evidence regarding the potential mechanisms through which the fear of cancer recurrence impacts death anxiety. It also contributes to our understanding of the role of experiential avoidance and the search for meaning in life in mitigating heightened death anxiety resulting from intense fear of cancer recurrence among cancer patients. By reducing experiential avoidance and fostering a sense of meaning in life, it may be possible to effectively alleviate both the fear of cancer recurrence and death anxiety experienced in cancer patients.

## Data Availability

The datasets used and/or analyzed during the current study available from the corresponding author on reasonable request.
